# The non-invasive serum biomarker soluble Axl accurately detects advanced liver fibrosis and cirrhosis

**DOI:** 10.1038/cddis.2017.554

**Published:** 2017-10-26

**Authors:** Katharina Staufer, Mirko Dengler, Heidemarie Huber, Rodrig Marculescu, Rudolf Stauber, Carolin Lackner, Hans-Peter Dienes, Danijel Kivaranovic, Christian Schachner, Markus Zeitlinger, Beatrix Wulkersdorfer, Peter Rauch, Gerhard Prager, Michael Trauner, Wolfgang Mikulits

**Affiliations:** 1 Division of Transplantation, Department of Surgery, Medical University of Vienna, Vienna, Austria; 2 Division of Gastroenterology and Hepatology, Department of Internal Medicine III, Medical University of Vienna, Vienna, Austria; 3 Department of Internal Medicine I, Institute of Cancer Research, Comprehensive Cancer Center Vienna, Medical University of Vienna, Vienna, Austria; 4 Department of Laboratory Medicine, Medical University of Vienna, Vienna, Austria; 5 Division of Gastroenterology and Hepatology, Department of Internal Medicine, Medical University of Graz, Graz, Austria; 6 Institute of Pathology, Medical University of Graz, Graz, Austria; 7 Clinical Institute of Pathology, Medical University of Vienna, Vienna, Austria; 8 Department of Statistics and Operations Research, University of Vienna, Vienna, Austria; 9 Department of Clinical Pharmacology, Medical University of Vienna, Vienna, Austria; 10 Candor Bioscience GmbH, Wangen im Allgäu, Germany; 11 Division of General Surgery, Department of Surgery, Medical University of Vienna, Vienna, Austria

## Abstract

Soluble Axl (sAxl) was recently shown to be strongly released into the blood during liver fibrogenesis and hepatocellular carcinoma suggesting sAxl as a biomarker of liver diseases. In this study we are the first to evaluate sAxl in human serum in comparison to Enhanced Liver Fibrosis (ELF) test and transient elastography (TE; Fibroscan) for its value to detect significant (F≥2), advanced fibrosis (F≥3), and cirrhosis (F4) in different liver disease etiologies and healthy controls. To properly determine the diagnostic accuracy of sAxl, a test cohort as well as a validation cohort was employed using liver biopsy as a reference method. Most notably, sAxl was confirmed to be an accurate biomarker of liver fibrosis and cirrhosis. Its accuracy was increased, if total serum albumin was added to build a sAxl/albumin ratio. Thereby an AUC of 0.763, 0.776, 0.826, and 0.832 was achieved corresponding to histological fibrosis stages F≥2, F≥3, F4 with liver biopsy as a reference method, and cirrhosis according to imaging techniques, respectively. With a cut-off of 1.29, a sensitivity, specificity, PPV, and NPV of 78.5%, 80.1%, 44%, 94.9% for the detection of cirrhosis was achieved. In comparison, ELF test and TE showed an AUC of 0.910, and 0.934, respectively, for the detection of cirrhosis. However, performance of TE was not possible in 14.4% of patients and both, ELF™ test and TE bear the disadvantage of high costs. In conclusion, the sAxl/albumin ratio is suggested as an accurate biomarker of liver fibrosis and cirrhosis. Due to its easy applicability and low costs it is suitable as screening parameter for significant to advanced liver fibrosis and cirrhosis, especially if TE is not available or not applicable.

Detection of advanced liver fibrosis and cirrhosis is crucial to support therapeutic decisions, determine surveillance intervals, and predict clinical outcome.^1, 2, 3^ Liver fibrosis has been shown to be the most significant predictor of liver-related mortality in several liver diseases.^4, 5^ A variety of serum-based biomarker panels, frequently combined with clinical parameters, such as body mass index (BMI), age, or the presence of diabetes, have been investigated in comparison to liver biopsy as a non-invasive, lower-cost alternative. All of these markers, like APR Index (APRI),^6, 7^ Enhanced Liver Fibrosis (ELF) test,^8, 9^ Fibrosis 4 (FIB4)-Index,^10^ Non-alcoholic fatty liver disease (NAFLD) fibrosis score,^11^ Cirrhosis Probability in Hepatitis C (Lok) index^12^ or FibroTest,^13, 14^ were analysed in various liver diseases, mostly associated with non-alcoholic fatty liver disease (NAFLD) and viral hepatitis showing areas under the receiver operating curve (AUROC) of 0.67 to 0.94 for the detection of significant fibrosis (F) (F≥2), or advanced fibrosis (F≥3), respectively.^3^ Furthermore, transient elastography (TE) has been extensively studied for its applicability in routine clinical use. In this context, Fibroscan (Echosens, Paris, France), Acoustic Radiation Forced Impulse (ARFI; Siemens Healthcare Diagnostics Inc., NY, USA), and Supersonic Shear Imaging (SSI) have been evaluated mainly in NAFLD,^15, 16^ viral hepatitis^17^ and alcoholic liver disease (ALD).^18^ Limitations of these techniques are identified for specific patient populations such as patients with acute hepatitis, congestive heart failure, obesity, ascites or patients after abdominal surgery possibly leading to intraabdominal adhesions and are associated with increased expenses.^3^ The optimal non-invasive biomarker for liver fibrosis includes high specificity and sensitivity, is independent of liver disease etiology and body mass index (BMI), is easily accessible and cost – effective.^3, 19^

Enhanced levels of soluble Axl (sAxl) in combination with the ligand of the Axl receptor, Gas6, have been detected in liver cirrhosis and suggested as a diagnostic tool.^20, 21, 22^ Recently, we reported that sAxl is an accurate biomarker for advanced fibrosis, cirrhosis and hepatocellular carcinoma (HCC) showing an AUC of 0.918 for the detection of biopsy-proven advanced fibrosis (F≥3), and an AUC 0.935 for the detection of F4 as compared to healthy controls.^23^ In the present study, we re-evaluated sAxl as a biomarker of liver fibrosis in comparison to ELF™ test and TE using liver biopsy as a reference method in a test and a validation cohort.

## Results

### Study population

In total, 392 (median age: 50.8y; male sex: 55%) patients were included. Of these, 361 patients showed chronic liver diseases, i.e. NAFLD, viral hepatitis (either chronic hepatitis B or C), autoimmune hepatitis (AIH,) cholestatic liver disease (CLD, primary sclerosing cholangitis [PSC] and primary biliary cholangitis [PBC]), or overlap syndrome, ALD, drug-induced liver injury (DILI), and cryptogenic cirrhosis (Table 1). The test cohort, which consisted of 276 patients with chronic liver disease and 31 healthy controls (Medical University of Vienna), was compared to a validation cohort (Medical University of Graz) of 85 patients. Both cohorts did not significantly differ in terms of age, sex, and BMI distribution. None of the patients suffered from significant heart failure. In 4.1% of patients (15/361), chronic kidney disease (CKD) was present. None of the patients had advanced stages of CKD (4 or 5). Detailed patient characteristics are displayed in Table 1.

Histopathological results were available in 311 of 392 study participants. F2, F3 and F4 were present in 20.9% (*n*=65), 10.3% (*n*=32) and 20.3% (*n*=63) of patients, respectively (Table 2a). According to imaging techniques, cirrhosis was present in 16.6% (*n*=65) of patients (Table 1) (confirmed by liver biopsy in 63/65, no liver biopsy available in 2/65, no cirrhosis present in 327 patients). The distribution of fibrosis grades among the different liver disease etiologies, as determined by liver biopsy, is given in Table 2b. Further, all results of liver function tests are displayed in Table 2c.

### sAxl levels depend on stage of liver fibrosis

**Figure 1:**
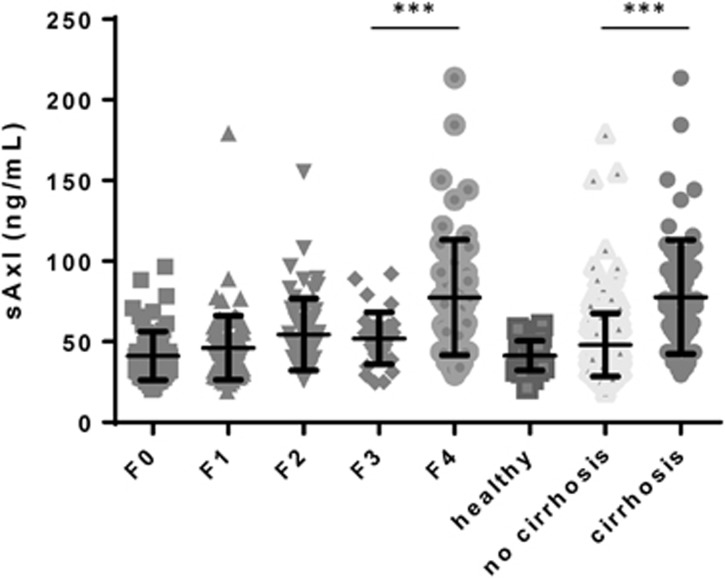
sAxl serum levels according to stage of fibrosis as well as presence of liver cirrhosis based on imaging methods. Statistical significant differences are expressed as asterisks: ***P<0.001

sAxl levels according to liver disease etiology are presented in Table 3. Notably, sAxl levels in patients with AIH/CLD were significantly higher in the validation cohort. This was explained by significantly higher sAxl levels found in patients with PBC which was caused by a selection bias due to small sample size since only 4 patients of the validation cohort were compared to 13 patients of the test cohort. Overall sAxl levels increased according to the stage of fibrosis (Figure 1). sAxl levels were significantly higher in patients with F4 as compared to F0 to F3 (median sAxl: 71.6, interquartile range (IQR) [53.91; 94.73] *versus* 43.4, IQR [35.52; 55.71]), as well as in patients with cirrhosis according to imaging compared to patients without cirrhosis as well as healthy controls (median sAxl: 72.06, IQR [54.06; 9.01] *versus* 43.3, IQR [35.85; 55.54] *versus* 40.2 IQR [34.2; 48.0]) (Figure 1). Together, these data reveal that sAxl levels do not significantly change at early stages of hepatic fibrosis (F0-F3) whereas sAxl is highly elevated in liver cirrhosis (F4) compared to advanced fibrosis (F3).

### Accuracy of sAxl to predict advanced liver fibrosis increases if combined with albumin

**Figure 2:**
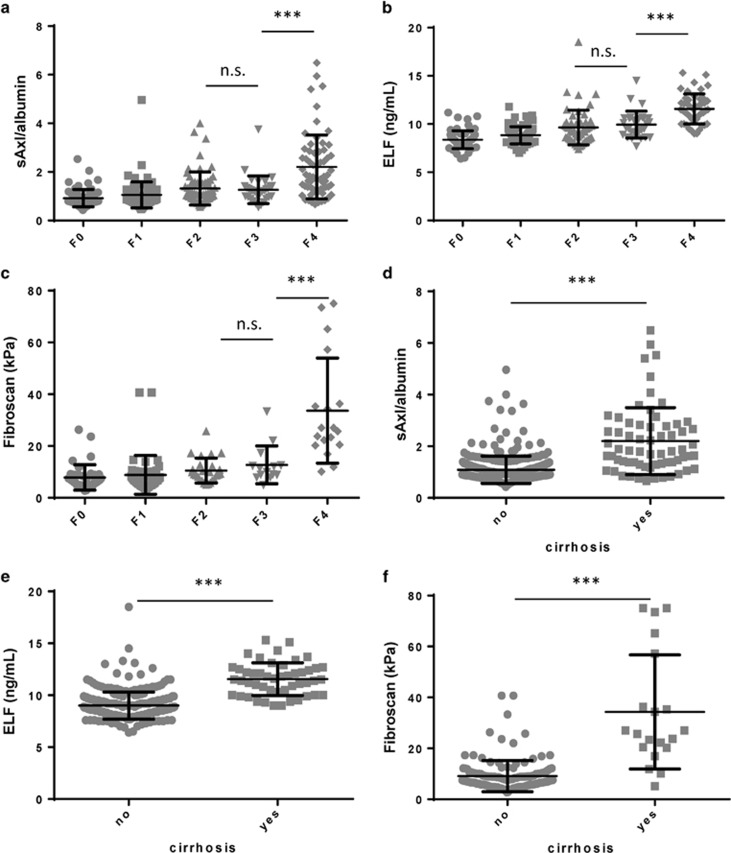
sAxl/albumin serum levels, ELF™ serum levels and Fibroscan results stratified according to liver biopsy (**a**–**c**) as well as stratified according to the presence of cirrhosis based on imaging test results (**d**–**f**). Statistical significant differences are expressed as asterisks: ***P<0.001

The AUC for the detection of F≥2, F≥3, and F4 compared to patients, and cirrhosis was 0.737, 0.749, 0.801, and 0.807, respectively (Table 4). Calculating a sAxl/albumin ratio increased the AUC to 0.763, 0.776, 0.826, and 0.831, respectively (Table 4, Supplementary Figure 1). sAxl/ albumin ratios according to fibrosis grade and cirrhosis are illustrated in Figures 2a and d.

### sAxl/albumin ratio has acceptable accuracy compared to ELF test and TE

ELF test and TE showed excellent diagnostic accuracy for the detection of liver fibrosis and cirrhosis. Data on their diagnostic performance are shown in Table 4 and Supplementary Figure 2. Yet, in terms of TE, 10.9% (*n*=8/73) patients of the test cohort, and 16.4 % (*n*=14/85) of the validation cohort had to be excluded due to unreliable measurements. Thus, in total 131 of 153 measurements were available for statistical analysis (Table 1).

**Figure 3:**
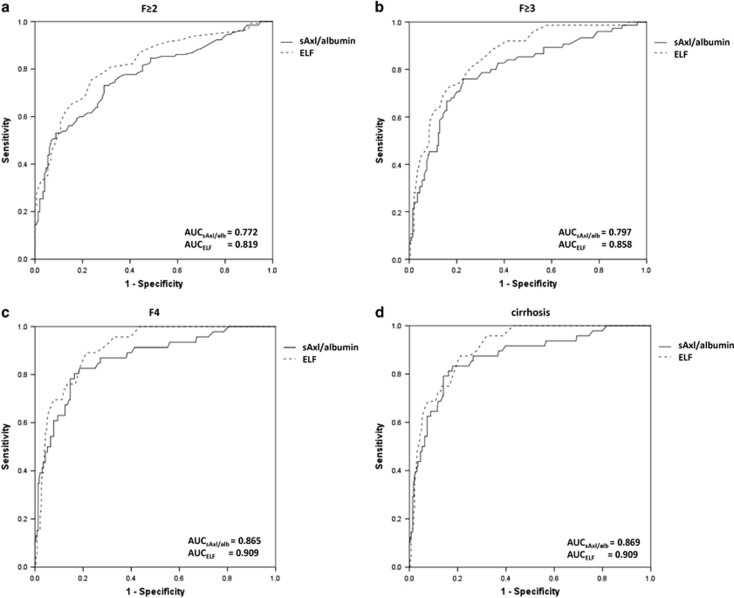
Comparison of the diagnostic accuracy displayed as area under the curve (AUC) of sAxl/albumin and ELF test for significant fibrosis (F≥2; (**a**), advanced fibrosis (F≥3; (**b**), fibrosis grade 4 (F4; (**c**), and liver cirrhosis according to imaging (**d**)

sAxl/albumin showed an acceptable accuracy for the detection of F≥2, F≥3, F4, and cirrhosis in comparison to ELF™ test (Figure 3, Table 4) as well as TE (Table 4). Spearman's correlation coefficients of sAxl with sAxl/albumin ratio, ELF test, and TE were 0.969 (*P*<0.001), 0.465 (*P*<0.001), and 0.215 (*P*=0.007), respectively. sAxl/albumin ratio correlated with ELF™ test and TE show a Spearman's correlation coefficient of 0.526 (*P*<0.001), and 0.240 (*P*=0.003), respectively.

### sAxl and sAxl/albumin ratio are independent of sex and BMI

sAxl and sAxl/albumin ratio were independent of sex (median sAxl [IQR]: male *versus* female: 46.1 [37.7; 58.1] *versus* 43.82 [35.48; 61.15], *P*=0.518; median sAxl/albumin [IQR]: 1.0 [0.83; 1.32] *versus* 1.02 [0.80; 1.50], *P*=0.683). Additionally, sAxl and sAxl/albumin ratio were independent of BMI category. In total, 35.7% (*n*=140/392) of patients were normal weight (BMI<25), 34.4% (*n*=135/392) of patients were overweight (25≤BMI≤30), and 29.8% (*n*=117/392) of patients fulfilled the WHO criteria of obesity (BMI ≥30).^24^ No significant differences were found between median sAxl [IQR] in normal weight, overweight and obese patients which was 45.0 ng/ml [37.9; 63.1], 47.1 ng/ml [38.4; 60.4], and 42.9 ng/ml [34.1; 55.1], respectively (*P*=0.056). Median sAxl/albumin ratio [IQR] in normal weight, overweight and obese patients was 1.01 [0.84; 1.43], 1.08 [0.85; 1.42], and 0.97 [0.77; 1.28], respectively (*P*=0.226).

## Discussion

sAxl was recently identified to be an excellent biomarker for early HCC^23, 25^ as well advanced fibrosis and liver cirrhosis.^23^ In the present study we aimed at investigating the diagnostic accuracy of sAxl as a biomarker for significant and advanced liver fibrosis in comparison to established non-invasive fibrosis markers such as ELF™ test and TE. The present study confirmed sAxl as an accurate biomarker of liver cirrhosis. Its diagnostic accuracy could be further increased by calculating a sAxl/albumin ratio achieving an AUC of 0.826 for the diagnosis of F4. At a cut-off of 1.29, a sensitivity of 77.8 and a specificity of 78.5 were reached. Notably, sAxl and sAxl/albuminratio was independent of sex and BMI.

Axl is a member of the TAM (Tyro3, Axl, Mer) subfamily of receptor tyrosine kinases and so far known to be involved in cancer development and mediation of chemoresistance.^26^ Besides affecting cell survival, proliferation, migration and angiogenesis, Axl additionally plays a role in the clearance of apoptotic cells, platelet aggregation and the regulation of pro-inflammatory cytokine production.^27, 28^ After proteolytic cleavage, the extracellular domain of Axl is released into the blood termed soluble Axl (sAxl).^29^ It has been shown to be elevated in serum or plasma in several diseases such as aortic aneurysm, heart failure, peripheral artery disease, CKD, systemic lupus erythematosus, rheumatoid arthritis, and preeclampsia.^30, 31, 32, 33, 34, 35^ Furthermore, our group was able to show that sAxl is predominantly released by liver myofibroblasts that are generally associated with liver fibrosis progression.^23^ These findings were in line with a recent study showing that Axl is involved in liver fibrosis progression in a Axl knockout mouse model proving that Axl signalling via Growth arrest-specific 6 is required for hepatic stellate cell activation.^20^

This is the first study to compare the diagnostic accuracy of sAxl as a non-invasive biomarker of liver fibrosis with ELF™ test and TE. The latter two are among the most intensively evaluated non-invasive tests for liver fibrosis and have been commercialized. The results of our study correspond well with previous reports on the diagnostic accuracy of ELF™ test and TE.^2, 6, 8^ However, both tests bear the disadvantage of high costs.^19^ Additionally, TE shows limitations in applicability since reliable results cannot be generated in up to 15.8% of patients mainly attributable to obesity, presence of ascites, or less operator experience.^8, 36, 37^ Therefore, in our cohort in total 14.4% of TE tests had to be excluded from analysis.

In contrast, sAxl is a stable serum marker that can easily be measured by enzyme-linked immunoassay (ELISA) at low costs.^38^ Recently, we reported a diagnostic accuracy with an AUC of 0.918 (cut-off 53.89 ng/ml) for the detection of F≥3, and an AUC of 0.935 (cut-off 54.0 ng/ml) for the detection of F4 in 190 chronic liver disease patients compared to healthy controls.^23^ In our present work we included 360 patients with chronic liver diseases as well as 31 healthy controls and we focused on discriminating significant to advanced fibrosis or cirrhosis from lower stages of fibrosis or healthy condition. sAxl thereby achieved an AUC of 0.801 (cut-off 52.98 ng/ml) in identifying patients with F4. Diagnostic accuracy was increased to an AUC of 0.826 (cut-off 1.29) for the detection of F4 (reference method liver biopsy), and an AUC of 0.832 (cut-off 1.29) for the detection of liver cirrhosis (reference method imaging) by adding total serum albumin to the model. Using the same cut-off of 1.29, the AUC for the detection of significant fibrosis (F≥2), advanced fibrosis (F≥3), and F4 was 0.763, 0.776, and 0.826, respectively. At the cut-off of 1.29, PPV and NPV for the detection of F4 were 46.2 and 93.9%, respectively, thereby reliably ruling out the presence of F4.

In this context, we have to consider that using liver biopsy as a reference method, although representing the gold standard, may automatically lead to an underestimation of non-invasive biomarkers (AUC <0.900) which is mostly due to sampling error and associated false negative results.^39, 40^

Furthermore, liver disease etiology has been previously shown to be a major factor influencing the performance of non-invasive fibrosis markers.^41^ Especially in cholestatic liver diseases, such as PBC and PSC, data on non-invasive fibrosis assessment are limited. Additionally, classical histologic scoring systems such as those according to Ludwig, fibrosis may be only partly addressed through stages 3 and 4 (ref. 42, 43). In our study sAxl/albumin ratio showed inconsistent cut-off levels within the cohort of AIH/CLD/Overlap patients with a cut-off of 3.66 for ≥F3, but a cut-off of 1.6 for the diagnosis of F4 maybe reflecting a bias of sample size since only 4 patients in this group were classified F3 in liver histology.

Study limitations include a possible underestimation of liver fibrosis as assessed by liver histology in CLD patients, and data on ELF™ test and TE in this patient cohort is limited. Consecutively, the accuracy calculation of sAxl or sAxl/albumin might be confounded translating into underdiagnosis of significant fibrosis. Moreover, our findings cannot be uncritically translated to patients with DILI, ALD or cryptogenic liver disease due to low patient numbers in these groups. Heart failure was excluded by the assessment of medical history and physical examination, but echocardiography was not routinely performed in all patients to exclude clinically inevitable or unknown heart failure. Therefore, sAxl levels might be increased due to unknown heart failure leading to false positive results in some patients.^34^ As CKD was reported to correlate with increased sAxl in plasma,^35^ we cannot exclude enhanced levels of sAxl due to mild CKD (stages 1-3) in 4.1% of our patients (15/361). However, median sAxl levels were lower in our patients with CKD than in patients without (48.09 *versus* 93.98 ng/ml). Neither the study by Batlle et al showing increased sAxl levels in heart failure,^34^ nor the study be Lee and colleagues reporting elevated sAxl levels in patients with CKD^35^ excluded liver diseases in their study cohorts. Further studies are warranted to investigate the true influence of heart failure and CKD on sAxl levels in liver disease patients.

In conclusion, sAxl/albumin ratio is an accurate marker of advanced liver fibrosis and cirrhosis in NAFLD and viral hepatitis. Due to its easy applicability and low costs it is suitable as screening parameter for significant to advanced liver fibrosis and cirrhosis, especially in case if TE is not available or not applicable. The diagnostic accuracy of sAxl in AIH and cholestatic liver diseases should be confirmed in different patient cohorts.

## Materials and methods

### Study population

Consecutive male and female patients with chronic liver disease as well as healthy volunteers were prospectively included in this study. Liver cirrhosis was either diagnosed by liver biopsy, liver imaging, i.e. ultrasound, computed tomography (CT) or magnetic resonance imaging (MRI), or both biopsy and imaging. Liver biopsy was evaluated by two independent pathologists (CL and HPD). Liver fibrosis was graded either according to Kleiner,^44^ Ludwig^42^ or METAVIR^45^ as appropriate. Blood sampling including liver function tests was performed on the day of liver biopsy or, if biopsy was not executed, within one week of liver imaging. Immediately after each blood withdrawal, one sample of whole blood was centrifuged according to a standardized protocol and stored as serum aliquots at −80 °C for later analysis of sAxl and ELF™ levels. In healthy volunteers liver disease was excluded by the thorough assessment of medical history, extensive laboratory tests as well as ultrasound. A test cohort was first investigated (Medical University of Vienna) followed by a validation cohort (Medical University of Graz) to confirm our findings. The medical history was thoroughly assessed in all patients and a complete physical examination was performed. Patients with acute infections, heart failure, immune-mediated diseases other than liver disease were not included in the study. Patients with CKD were categorized into CKD stages 1 to 5 (ref. 46). The study was conducted in accordance with the guidelines of the Declaration of Helsinki (1964, including current revisions) and GCP Guidelines after approval of the ethics committee of the Medical University of Vienna and Graz. All patients signed a written informed consent prior to study inclusion.

### Commercialized liver fibrosis tests

ELF test was measured in serum on the ADVIA Centaur Immunoassay system according to the manufacturer’s protocol (Siemens Healthcare Diagnostics Inc., NY, USA). TE using Fibroscan® (Echosens, Paris, France) was performed in fasted state and according to the manufacturer's instructions. Results showing <10 valid measurements, a success rate <60% or an IQR>30% were excluded from the analysis.

### Enzyme-linked immunosorbent assay (ELISA)

sAxl was measured in serum by solid phase sandwich ELISAs according to manufacturer’s protocol (R&D Systems, Minneapolis, USA) including modifications for optimization of the assay as published previously.^23, 38^

### Statistical analysis

Quantitative variables were expressed as median with interquartile range (IQR) and compared by Mann-Whitney U-tests. Qualitative variables were described using absolute and relative frequencies and were compared by Chi-Square- or Fisher's exact tests, as appropriate. Comparison of more than two groups was done by the use of the Kruskal Wallis test. The Spearman's correlation coefficient was used in order to assess correlations between all utilized non-invasive tests.The accuracy of sAxl was assessed by AUC to predict (1) significant fibrosis (≥F2), (2) advanced fibrosis (≥F3), (3) F4, and (4) liver cirrhosis according to imaging as (A) a single marker and in combination with (C) serum albumin levels (mg/dl; expressed as sAxl/albumin ratio). In order to identify optimal cut-off values, the Youden index was calculated.^47^ Additionally, sensitivity, specificity, PPV, and NPV were computed. *P*-values <0.05 were considered statistically significant. Statistical analysis was performed using IBM Statistics SPSS 24.0 (IBM Corp., Armonk, NY).

## Publisher’s Note

Springer Nature remains neutral with regard to jurisdictional claims in published maps and institutional affiliations.

## Figures and Tables

**Table 1:** Patient characteristics

	**All patients *N*=392**	**Test cohort *N*=307**	**Validation cohort *N*=85**	***P*-value**
Age (y); median (IQR)	50.8 (38.3;59.5)	50.4 (37.1;59.8)	52.0 (43.0;58.5)	0.207
Male sex; % (*n*)	55.0 (216)	56.7 (174)	49.4 (42)	0.233
BMI; median (IQR)	26.5 (23.7;31.3)	26.6 (23.7;33.3)	26.0 (23.5;28.5)	0.074
				
*Liver disease etiology; %* (n)				
NAFLD	43.6 (171)	45.3 (139)	37.6 (32)	0.040
Viral hepatitis	21.9 (86)	15.6 (48)	44.7 (38)	<0.001
AIH/CLD/Overlap	18.1 (71)	19.2 (59)	14.1 (12)	0.141
PBC/PSC	8.6 (34)	9.8 (30)	4.7 (4)	0.349
AIH/Overlap	9.4 (37)	9.4 (29)	9.4 (8)	1.000
DILI	2.0 (8)	2.0 (6)	2.4 (2)	1.000
ALD	4.1 (16)	5.2 (16)	0.0 (0)	0.016
Cryptogenic	2.3 (9)	2.6 (8)	1.2 (1)	0.692
Healthy controls	7.9 (31)	10.1 (31)	0.0 (0)	n.d.
*Cirrhosis according to imaging; %* (n)	16.6 (65)	16.9 (52)	15.3 (13)	0.718
US only	63.1 (41)	53.8 (28)	100 (13)	
US + CT	15.4 (10)	19.2 (10)	0 (0)	
US + MRI	13.8 (9)	17.3 (9)	0 (0)	
US + CT + MRI	7.7 (5)	9.6 (5)	0 (0)	
Liver biopsy; % (*n*)	79.3 (311)	73.6 (226)	100 (85)	n.d.
sAxl; % (*n*)	100 (392)	100 (307)	100 (85)	n.d.
ELF test, % (*n*)	84.7 (332)	83.4 (256)	89.4 (76)	n.d.
Fibroscan; % (*n*)	33.4 (131)	21.2 (65)	77.6 (66)	n.d.

Abbreviations: AIH, autoimmune hepatitis; ALD, alcoholic liver disease; CT, computed tomography; CLD, cholestatic liver disease; DILI, drug-induced liver injury; IQR, interquartile range; MRI, magnetic resonance imaging; n.d., not determined; NAFLD, non-alcoholic fatty liver disease; PBC, primary biliary cholangitis; PSC, primary sclerosing cholangitis; US, ultrasound

**Table 2a:** Fibrosis grades based on liver biopsy

	**All patients *N*=311/392**	**Test cohort *N*=226/307**	**Validation cohort *N*=85/85**	***P*-value**
*Fibrosis stage; %* (n)				
0	21.9 (68)	20.4 (46)	25.9 (22)	0.155
1	26.7 (83)	26.5 (60)	27.1 (23)	0.987
2	20.9 (65)	21.7 (49)	18.8 (16)	0.517
3	10.3 (32)	9.3 (21)	12.9 (11)	0.379
4	20.3 (63)	22.1 (50)	15.3 (13)	0.155

Liver biopsy was available in 311 of 392 patients

**Table 2b:** Distribution of fibrosis grades stratified according to liver disease etiology (*n*=311)

**Liver disease etiology; % (*n*)**	**F0-1**	**F2**	**F3**	**F4**
NAFLD	62.0 (93)	13.3 (20)	11.3 (17)	13.3 (20)
Viral hepatitis	36.0 (31)	26.7 (23)	12.8 (11)	24.4 (21)
AIH/CLD/Overlap	18.6 (8)	46.5 (20)	9.3 (4)	25.6 (11)
DILI	87.5 (7)	12.5 (1)	0.0 (0)	0.0 (0)
ALD	0.0 (0)	10.0 (1)	0.0 (0)	90.0 (9)
Cryptogenic	77.8 (7)	0.0 (0)	0.0 (0)	22.2 (2)

**Table 2c:** Laboratory parameters of patients with chronic liver diseases

**Laboratory parameter**	**All patients *n*=360/392**	**Test cohort *n*=275/307**	**Validation cohort *n*=85/85**	***P*-value**
AST (U/ml) median (IQR)	42.5 (29.0;68.0)	39.0 (27.0;64.0)	53.0 (34.0;75.0)	0.004
ALT (U/ml); median (IQR)	52.0 (33.0;93.5)	50.5 (31.0;91.0)	59.0 (40.0;115.5)	0.057
GGT (U/ml); median (IQR)	80.0 (38.0;186.0)	80.0 (37.0;208.0)	84.0 (45.5;166.0)	0.817
Albumin (mg/dl); median (IQR)	44.0 (41.0; 46.4)	43.5 (39.9;46.3)	45.0 (43.0;47.0)	0.016
Total bilirubin (mg/dl); median (IQR)	0.7 (0.5;1.0)	0.7 (0.5;1.1)	0.64 (0.46;0.86)	0.033
Platelet count (G/L); median (IQR)	221.5 (180.0;276.8)	224.0 (181.0;285.0)	212.0 (172.0;250.5)	0.106
MELD score; median (IQR)	7.2 (6.4;8.5)	7.5 (6.4;8.5)	6.9 (6.4;7.8)	0.049

**Table 3:** Results of non-invasive fibrosis assessment

		**All patients *n*=392**	**Test cohort *n*=307**	**Validation cohort *n*=85**	***P*-value**
	Liver disease etiology				
sAxl level (ng/ml); median (IQR)	Liver disease, *n*=361	45.7 (37.2;60.8)	44.0 (36.1;59.1)	53.0 (42.3;70.1)	0.002
	NAFLD	41.2 (33.8;52.8)	40.7 (33.0;50.8)	43.7 (36.4;57.5)	0.076
	Viral hepatitis	56.6 (43.3;71.7)	56.5 (43.0;77.3)	57.3 (43.4;70.8)	0.945
	AIH/CLD/Overlap	45.6 (37.6;70.2)	43.7 (36.2;60.3)	77.4 (51.9;83.0)	0.001
	DILI	47.9 (34.5;75.4)	62.2 (36.3;103.0)	n.d.	n.d.
	ALD	63.5 (47.4;92.1)	63.5 (47.4;92.1)	n.d.	n.d.
	Cryptogenic	51.2 (38.7;67.2)	50.1 (38.1;62.7)	n.d.	n.d.
	Healthy controls, *n*=31	40.2 (34.2;48.0)	40.2 (34.2;48.0)	n.d.	n.d.
sAxl/albumin; median (IQR)	Liver disease, *n*=361	(0.82;1.38)	1.00 (0.80;1.39)	1.21 (0.89;1.56)	0.004
	NAFLD	0.92 (0.75;1.14)	0.92 (0.75;1.13)	0.93 (0.80;1.32)	0.322
	Viral hepatitis	1.29 (0.97;1.69)	1.32 (0.96;1.94)	1.28 (0.98;1.58)	0.537
	AIH/CLD/Overlap	1.07 (0.86;1.56)	0.99 (0.84;1.40)	1.94 (1.20;2.46)	0.002
	DILI	1.16 (0.78;1.78)	1.50 (0.86;2.63)	n.d.	
	ALD	1.72 (1.07;2.59)	1.72 (1.07;2.59)	n.d.	
	Cryptogenic	1.11 (0.86;1.70)	1.08 (0.85;1.68)	n.d.	
	Healthy controls, *n*=31	0.85 (0.78;1.04)	0.85 (0.78;1.04)	n.d.	
ELF^TM^ test (ng/ml); median (IQR)	Liver disease, *n*=305	9.0 (8.3;10.1)	9.2 (8.4;10.5)	8.7 (8.1;9.5)	0.012
	NAFLD, *n*=162	8.7 (8.1;9.5)	8.8 (8.2;9.7)	8.3 (7.9;9.0)	0.009
	Viral hepatitis, *n*=66	9.3 (8.6;10.3)	9.9 (8.8;11.0)	9.0 (8.6;9.8)	0.033
	AIH/CLD/Overlap, *n*=62	9.4 (8.3;10.8)	9.4 (8.3;10.7)	9.0 (8.3;11.6)	0.781
	DILI	n.d.	n.d.	n.d.	n.d.
	ALD, *n*=13	11.5 (10.1;12.4)	11.5 (10.1;12.4)	n.d.	n.d.
	Cryptogenic	n.d.	n.d.	n.d.	n.d.
	Healthy controls, *n*=27	8.7 (8.0;9.3)	8.7 (8.0;9.3)	n.d.	n.d.
Fibroscan (kPa); median (IQR)	Liver disease, *n*=131	7.9 (5.8;11.8)	8.0 (6.0;11.6)	7.8 (5.4;11.8)	0.715
	NAFLD, *n*=75	8.1 (6.0;10.6)	8.4 (6.2;11.4)	7.6 (5.8;10.5)	0.841
	Viral hepatitis, *n*=33	8.8 (4.9;12.6)	n.d.	8.8 (4.9;12.5)	n.d.
	AIH/CLD/Overlap, *n*=18	7.2 (5.9;10.1)	6.4 (5.8;10.4)	7.8 (6.9;10.0)	0.328
	DILI	n.d.	n.d.	n.d.	n.d.
	ALD	n.d.	n.d.	n.d.	n.d.
	Cryptogenic	n.d.	n.d.	n.d.	n.d.
	Healthy controls, *n*=0	n.d.	n.d.	n.d.	n.d.

Results of non-invasive fibrosis markers were compared by Mann Whitney U – test. n.d., not determined

**Table 4:** Accuracy of sAxl, sAxl/albumin ratio, ELF™ test and transient elastography (Fibroscan®) for the detection of significant fibrosis (≥F2), advanced fibrosis (≥F3), F4 and cirrhosis according to imaging

**Fibrosis/cirrhosis**	**AUC (95%CI)**	**Sensitivity (%)**	**Specificity (%)**	**PPV**	**NPV**	**Youden's Index**	**Cut-off**
*sAxl*							
All patients							
F≥2	0.737	59.4	80.2	73.1	68.6	0.396	52.78
F≥3	0.749	70.5	74.0	51.5	86.5	0.445	52.78
F4	0.801	77.8	71.9	38.9	93.4	0.497	52.98
Cirrhosis	0.807	78.5	72.8	36.4	94.4	0.512	52.98
NAFLD							
F≥2	0.692	49.1	83.9	65.1	72.9	0.330	48.96
F≥3	0.747	62.2	86.7	60.5	87.5	0.489	52.78
F4	0.788	70.0	83.1	38.9	94.7	0.531	52.97
Cirrhosis	0.776	70.0	82.1	34.1	95.4	0.521	52.97
Viral hepatitis							
F≥2	0.697	69.1	67.7	79.2	55.3	0.368	54.01
F≥3	0.718	78.1	57.4	52.1	81.6	0.355	54.00
F4	0.788	71.4	80.0	53.6	89.7	0.514	65.32
Cirrhosis	0.788	71.4	80.0	53.6	89.7	0.514	65.32
AIH/CLD/Overlap							
F≥2	0.754	60.0	87.5	33.3	47.5	0.475	52.22
F≥3	0.640	60.0	67.9	50.0	76.0	0.279	60.32
F4	0.696	72.7	68.8	44.4	88.0	0.415	60.32
Cirrhosis	0.760	75.0	78.0	40.9	93.9	0.530	60.32
							
*sAxl/albumin*							
All patients							
F≥2	0.763	54.4	88.1	81.3	68.3	0.425	1.29
F≥3	0.776	65.3	81.0	53.1	87.4	0.463	1.29
F4	0.826	77.8	78.5	46.2	93.9	0.562	1.29
Cirrhosis	0.831	78.5	79.5	44.0	94.9	0.580	1.29
NAFLD							
F≥2	0.712	61.4	78.5	63.6	76.8	0.399	1.03
F≥3	0.774	62.2	92.0	71.9	88.1	0.542	1.19
F4	0.798	65.0	92.3	56.5	94.5	0.573	1.36
Cirrhosis	0.789	65.0	91.4	50.0	95.2	0.564	1.36
Viral hepatitis							
F≥2	0.736	54.5	87.1	88.2	51.9	0.416	1.42
F≥3	0.750	53.1	90.7	77.3	76.6	0.439	1.69
F4	0.823	71.4	89.2	68.2	90.6	0.607	1.69
Cirrhosis	0.823	71.4	89.2	68.2	90.6	0.607	1.69
AIH/CLD/Overlap							
F≥2	0.750	60.0	87.5	95.5	33.3	0.475	1.22
F≥3	0.657	33.0	96.4	83.0	73.0	0.298	3.66
F4	0.739	63.6	75.0	46.7	85.7	0.386	1.60
Cirrhosis	0.805	75.0	79.7	42.9	94.0	0.547	1.47
							
*ELF™test*							
All patients							
F≥2	0.819	75.4	76.4	73.7	77.9	0.517	9.2
F≥3	0.858	72.0	83.7	64.3	89.2	0.557	9.9
F4	0.909	89.1	78.4	46.6	97.4	0.675	9.8
Cirrhosis	0.909	87.5	78.9	42.4	97.4	0.664	9.8
NAFLD							
F≥2	0.831	77.4	80.7	70.7	85.5	0.580	9.2
F≥3	0.876	90.9	75.0	52.6	96.4	0.659	9.2
F4	0.929	93.8	84.8	44.1	99.1	0.785	9.7
Cirrhosis	0.936	93.8	86.3	42.9	99.2	0.801	9.7
Viral hepatitis							
F≥2	0.763	82.9	64.0	79.1	69.6	0.469	8.9
F≥3	0.792	56.5	90.7	76.5	79.6	0.472	10.2
F4	0.813	100	47.2	31.7	100	0.472	9.0
Cirrhosis	0.813	100	47.2	31.7	100	0.472	9.0
AIH/CLD/Overlap							
F≥2	0.747	50.0	100	100	33.0	0.500	10.8
F≥3	0.795	81.8	79.2	64.3	90.5	0.610	10.8
F4	0.872	100	80.8	64.3	100	0.808	10.8
Cirrhosis	0.898	90.0	88.5	60.0	97.9	0.785	10.8
							
*Fibroscan®*							
All patients							
F≥2	0.850	67.0	86.0	77.3	77.9	0.524	9.4
F≥3	0.890	76.0	88.0	66.7	92.0	0.639	11.1
F4	0.970	88.0	95.0	75.0	98.0	0.834	16.6
Cirrhosis	0.930	84.0	96.0	76.2	97.3	0.799	16.6
NAFLD							
F≥2	0.853	81.8	74.5	58.1	90.5	0.563	8.9
F≥3	0.935	90.9	80.6	45.5	98.0	0.716	10.1
F4	0.935	100	78.5	36.4	100	0.785	10.1
Cirrhosis	0.937	100	79.1	36.4	100	0.791	10.1
Viral hepatitis							
F≥2	0.760	85.0	100	100	81.2	0.850	8.9
F≥3	0.887	92.3	75.0	70.6	93.8	0.673	8.8
F4	1.000	100	100	100	100	1.000	18.8
cirrhosis	1.000	100	100	100	100	1.000	18.8
AIH/CLD/Overlap							
F≥2	n.d.	n.d.	n.d.	n.d.	n.d.	n.d.	n.d.
F≥3	n.d.	n.d.	n.d.	n.d.	n.d.	n.d.	n.d.
F4	n.d.	n.d.	n.d.	n.d.	n.d.	n.d.	n.d.
cirrhosis	1.000	100	100	100	100	1.000	23.1

Abbreviation: n.d., not determined
