# Supplementary figures and images for: The non-invasive serum biomarker soluble Axl accurately detects advanced liver fibrosis and cirrhosis
Source: Cell Death Dis. 2017 Oct 26;8(10):e3135–. doi: 10.1038/cddis.2017.554 (PMC5680921; doi:10.1038/cddis.2017.554)

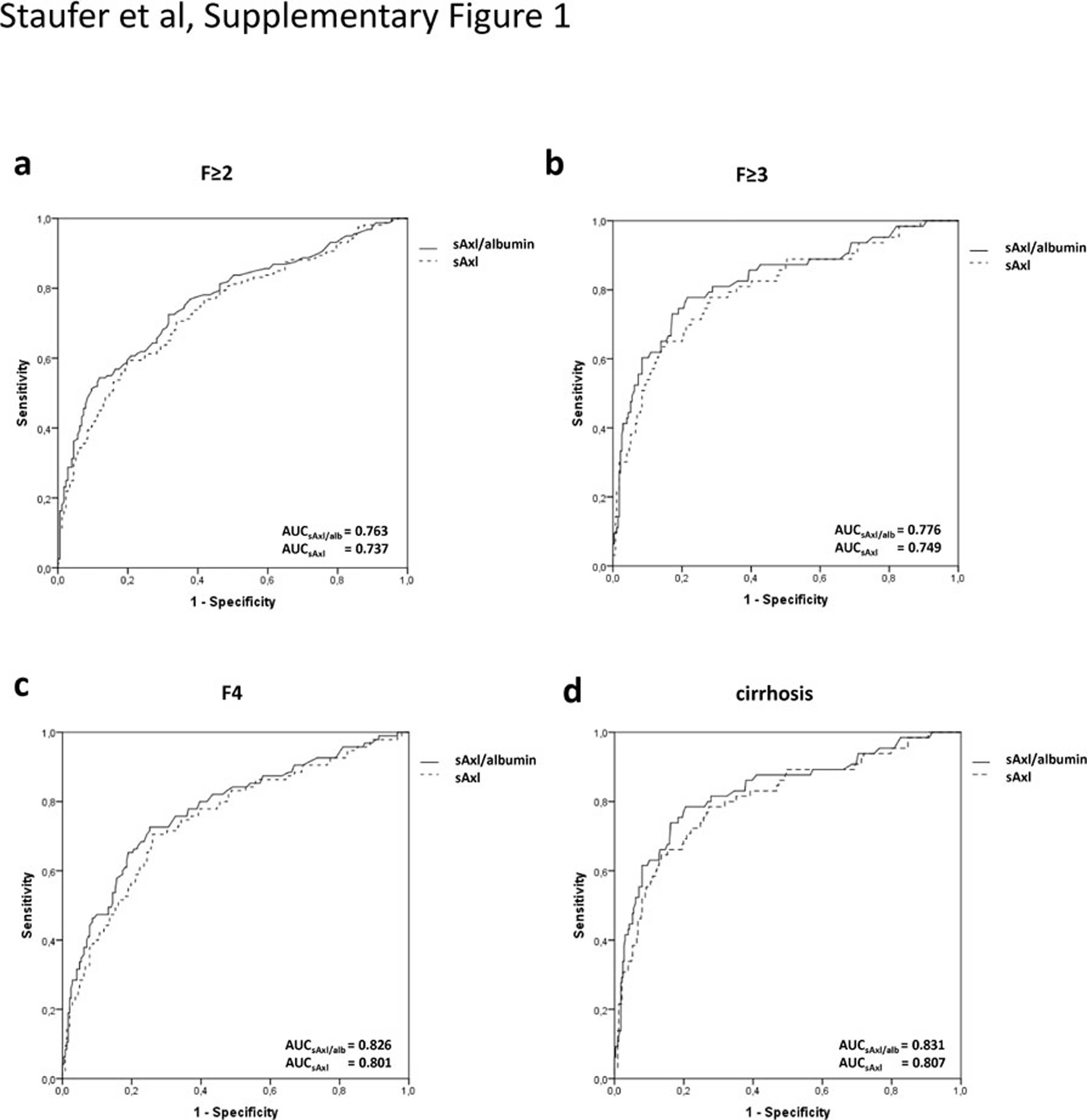

Supplement: Supplementary Figure 1 [file cddis2017554x1.tif]

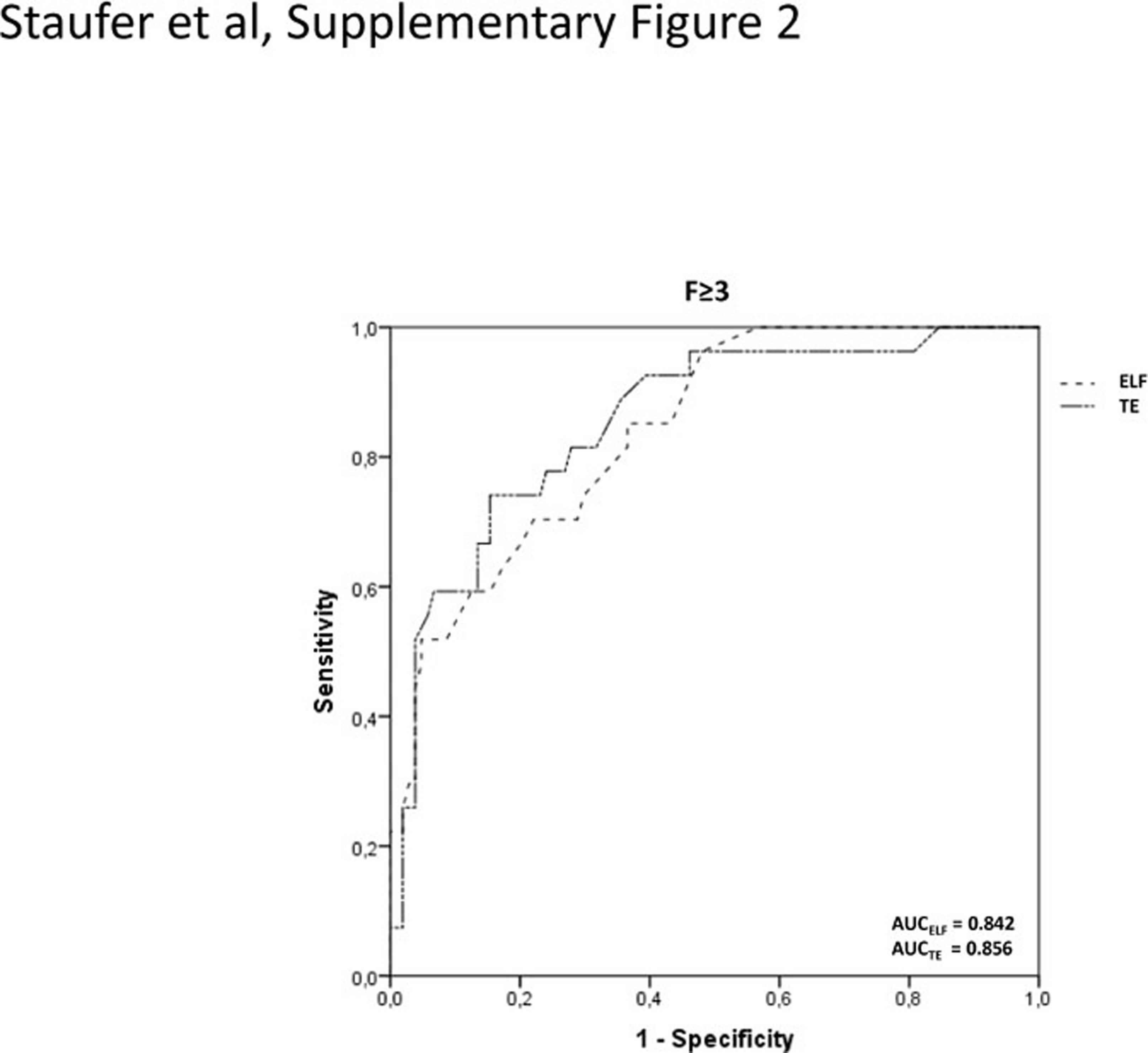

Supplement: Supplementary Figure 2 [file cddis2017554x2.tif]
