# Supplementary material for: The non-invasive serum biomarker soluble Axl accurately detects advanced liver fibrosis and cirrhosis
Source: Cell Death Dis. 2017 Oct 26;8(10):e3135–. doi: 10.1038/cddis.2017.554 (PMC5680921; doi:10.1038/cddis.2017.554)
Supplement: Supplementary Figure Legends [file cddis2017554x3.docx]

**Supplementary Figure Legends**

**Supplementary Figure 1** The accuracy of sAxl is increased by calculating a sAxl/albumin ratio. To calculate the sAxl/albumin ratio total serum albumin in mg/dL was used. Displayed are areas under the Receiver Operating Characteristic (ROC) curve for the detection of significant fibrosis (F≥2; panel A), advanced fibrosis (F≥3; panel B), fibrosis grade 4 (F4; panel C), and liver cirrhosis according to imaging (panel D).

**Supplementary Figure 2** ELF™ serum levels (AUC: 0.842) in comparison to Fibroscan® show similar accuracy in the detection of F≥3. AUC, area under the ROC curve.
